# Macrophage phenotype in sepsis immunosuppression

**DOI:** 10.1186/cc14124

**Published:** 2015-03-16

**Authors:** E Theodorakis, E Diamantaki, C Tsatsanis, D Georgopoulos, K Vaporidi

**Affiliations:** 1 University of Crete, School of Medicine, Heraklio, Greece

## Introduction

Sepsis is followed by profound, yet poorly characterized, innate immune system suppression. While low monocyte HLA-DR expression is observed in septic patients, its clinical significance has not been established [1]. *In vitro*, repeated LPS stimulation induces a tolerant or M2 macrophage phenotype, characterized by decreased cytokine production [2], which could contribute to sepsis immunosuppression. The present study examines macrophage phenotype in a mouse model and in patients with sepsis immunosuppression.

## Methods

Sepsis was induced in C57Bl6 mice by cecal ligation and puncture (CLP) followed by intratracheal instillation of *Pseudomonas aeruginosa*. Bronchoalveolar lavage fluid (BALF), cells and serum, collected 12 hours after lung infection, were analyzed for bacterial load, cytokine levels and the classical M1 marker, iNOS. Peripheral blood monocytes isolated from septic adult patients admitted to the ICU on the 1st and 7th day after admission were analyzed by flow cytometry for the expression of HLA-DR and CD86 (co-stimulatory molecule and M1 marker), and for the M2 markers, CD163 and CD206. Additional blood samples from patients and healthy volunteers were exposed *ex vivo *to LPS prior to isolation and analysis of monocyte markers.

## Results

CLP-induced sepsis resulted in immunosuppression in mice, indicated by higher BALF bacterial load after infection in CLP than in sham-operated mice, and more severe injury on histology. Serum cytokines TNF and MIP2 were greater in CLP than in sham-operated mice. Although recruitment of CD11c^+ ^alveolar macrophages post infection was threefold greater in CLP than in sham-operated mice, those macrophages expressed 40% lower levels of iNOS. Evidence of sepsis immunosuppression was present in most patients on the 7th day after ICU admission. Low expression of CD86 and/or HLA-DR was observed in 71% of patients, and increased expression of M2 markers in 15% of patients. Upon LPS stimulation the normal decrease in M2 markers was absent in all patients on day 1, and partially restored in 50% of patients on day 7.

## Conclusion

Sepsis is associated with decreased monocyte expression of M1 markers and increased expression of M2 markers in septic mice and critically ill patients. Therefore, in addition to decreased HLA-DR expression, M2 macrophage polarization appears to be a component of sepsis-induced monocyte dysfunction, and should be considered for immune monitoring and targeted intervention.

## Acknowledgements

Supported by GSRT research grant ExcellenceII-4620.
